# Bacterial Attachment to Polymeric Materials Correlates with Molecular Flexibility and Hydrophilicity

**DOI:** 10.1002/adhm.201400648

**Published:** 2014-12-09

**Authors:** Olutoba Sanni, Chien-Yi Chang, Daniel G Anderson, Robert Langer, Martyn C Davies, Philip M Williams, Paul Williams, Morgan R Alexander, Andrew L Hook*

**Affiliations:** 1 School of Pharmacy University of Rome, Tor VergataVia Della Ricerca Scientifica 1, Rome, 00133, Italy; 2 The Centre for Bacterial Cell Biology, Medical School, Newcastle UniversityNewcastle upon Tyne, NE2 4AX, UK; 3 Interdisciplinary Computing and Complex BioSystems (ICOS) research group, School of Computing Science, Newcastle UniversityNewcastle upon Tyne, NE1 7RU, UK; 4 Department of Chemical Engineering, Institute for Medical Engineering and Science, Harvard-MIT Division of Health Sciences and Technology, David H. Koch Institute for Integrative Cancer Research, Massachusetts Institute of Technology500 Main Street, Cambridge, MA, 02139, USA; 5 Laboratory of Biophysics and Surface Analysis, School of Pharmacy, University of NottinghamNottingham, NG72RD, UK; 6 School of Life Sciences, Centre for Biomolecular Sciences, University of NottinghamNottingham, NG72RD, UK

**Keywords:** low-fouling, molecular descriptors, polymer microarrays, *Pseudomonas aeruginosa*, ion mass spectrometry

## Abstract

A new class of material resistant to bacterial attachment has been discovered that is formed from polyacrylates with hydrocarbon pendant groups. In this study, the relationship between the nature of the hydrocarbon moiety and resistance to bacteria is explored, comparing cyclic, aromatic, and linear chemical groups. A correlation is shown between bacterial attachment and a parameter derived from the partition coefficient and the number of rotatable bonds of the materials' pendant groups. This correlation is applicable to 86% of the hydrocarbon pendant moieties surveyed, quantitatively supporting the previous qualitative observation that bacteria are repelled from poly(meth)acrylates containing a hydrophilic ester group when the pendant group is both rigid and hydrophobic. This insight will help inform and predict the further development of polymers resistant to bacterial attachment.

## 1. Introduction

Novel materials that are able to resist bacterial attachment are highly attractive for numerous applications, including preventing medical-device-associated infections and marine fouling of ship hulls. Following attachment to a surface, bacteria form biofilms within which they are much more resistant to antimicrobials and host defenses than individual, planktonic (suspended) bacterial cells.[1] Biofilms are communities of bacteria that exist within an extracellular polymeric substance (EPS) composed of polysaccharides, proteins, and nucleic acids.[2] To prevent biofilm formation at the earliest possible stage, the surface of medical devices could be engineered to prevent bacterial attachment. There are three types of synthetic polymers reported to reduce bacterial attachment without involving a component designed to specifically kill bacteria: these are poly(ethylene glycol) (PEG)-based polymers,[3] zwitterionic polymers,[4,5] and weakly amphiphilic poly(meth)acrylates.[6,7] The first two material classes are highly hydrophilic and the mechanism for the anti-fouling behavior of these polymers is thought to be associated with the exclusion of biomolecules from the surface due to its association with water. The third class of materials was recently discovered using a high-throughput materials discovery methodology.[6,7] The (meth)acrylate polymers that best resisted attachment of a range of pathogens in a 3-d immersion test were those with cyclic and aromatic hydrocarbon pendent groups.[7] In attempting to rationalize the anti-attachment mechanism involved, it was noted that a range of surface properties did not correlate with bacterial attachment, including wettability, elemental and functional composition from X-ray photoelectron spectroscopy (XPS) and topography.[7] The one approach that did yield some predictive power was the structurally rich surface mass spectrometry technique of time-of-flight secondary ion mass spectrometry (ToF-SIMS). Structurally, it is notable that the bacterial attachment resistance of these materials contrasts with the extensive biofilm formation observed on polystyrene, which is structurally analogous to some of the hits reported with the omission of an ester group.[7] These polymers, which were classified as weakly amphiphilic, did not alter growth profiles of planktonic bacteria, indicating that their mechanism of action involves attachment prevention rather than killing; nor was media incubated in their presence cytotoxic to mammalian cells, indicating significant potential as a material for reducing medical-device-centered infections.[7] These materials were able to resist both Gram-positive (*Staphylococcus auerus*) and Gram-negative (*Pseudomonas aeruginosa*, *Escherichia coli*) bacterial attachment. Structural modeling using a nonlinear Bayesian neural net of bacterial attachment with molecular descriptors revealed a number of descriptors associated with hydrophobicity (e.g., the number of hydrogen bond acceptors on nitrogen, calculated log octanol/water partition coefficient (*c*log *P*), number of OH groups, number of methyl and methylene groups) or molecular shape (e.g., number of tetrahedral atomic stereocenters, the molecular eccentricity) to be relevant to *P. aeruginosa* attachment.[8] In particular, the descriptor *c*log P is a well-established measure of the hydrophilicity of a compound and has been widely used for estimating the permeability and distribution of drugs within the body.

**Figure 1:**
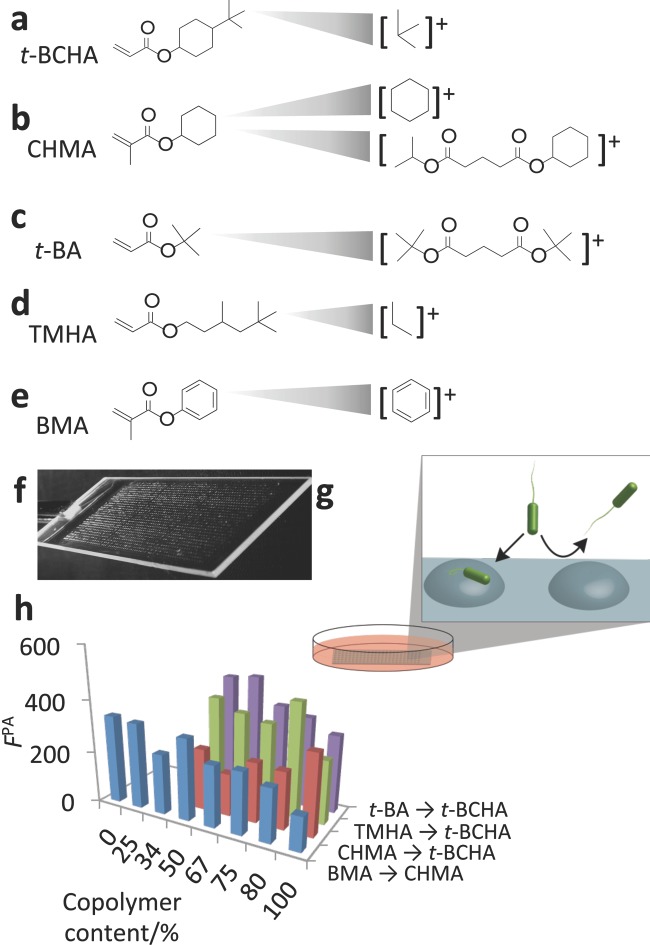
a–e) Chemical structures of monomers used in study and the SIMS secondary ions specific to each monomer. f) Image of polymer microarray used to perform bacterial attachment assay. g) After preparation the polymer, microarray was incubated with *P. aeruginosa* for 72 h in RPMI 1640 media to assess bacterial attachment, shown schematically. h) Bacterial attachment was quantified by measuring the fluorescence from GFP-transformed *P. aeruginosa* (*F*^PA^), shown for the four polymer series produced from the five monomers.

In this paper, we fabricate a microarray of materials designed specifically to probe the structure–performance relationships and used the technique of ToF-SIMS to search for structural correlations with the bacterial response. The mono­mer *tert-*butyl cyclohexyl acrylate (*t-*BCHA **Figure** **1**a) was one of five “hits” that successfully produced polymers resisting bacterial attachment in the initial report of the amphiphilic polymers.[7] Taking this monomer as an example, it has a number of moieties (*tert-*butyl, cyclohexyl, and ester) that may contribute to resistance to bacterial attachment. In this study, we mixed *t-*BCHA with structural homologues to form a series of copolymers in order to probe the role of specific functional groups for preventing bacterial attachment. To achieve this rapidly, we employed the polymer microarray format.[9–11] Polymer microarrays have to date largely been used as a material discovery tool,[12–14] whereby the polymer library expressed on the microarray is selected in order to maximize the diversity of materials.[15] This approach is relevant when the underlying cell–material interaction is not well understood.[16,17] However, where a hypothesis exists the members of the polymer library expressed on a microarray can be judiciously chosen to explore that hypothesis. The present work explores the hypothesis that a more rigid hydrocarbon pendant groups on a polyacrylate will improve resistance to bacterial attachment. We demonstrate that when considered together, material hydrophobicity and molecular stiffness of the pendant group correlate with bacterial attachment to polymerized (meth)acrylates with hydrocarbon pendant groups.

## 2. Results

### 2.1 Assessment of Surface Chemistry and Bacterial Attachment

A library of copolymers was formed where *t-*BCHA was mixed sequentially with three monomers: cyclohexyl methacrylate (CHMA), *tert-*butyl acrylate (*t-*BA), and 3,5,5-trimethylhexyl acrylate (TMHA) (Figure 1b–d). The co-monomers were mixed with *t-*BCHA at ratios of 1:0, 4:1, 3:1, 2:1, and 1:1 to form statistical copolymers, whereby the sequence of monomers follows the statistics of the monomer feeds. To compare cyclic and aromatic moieties, CHMA was mixed sequentially with benzyl methacrylate (BMA, Figure 1e). On the slides, three replicates of each material were printed. The resulting array (Figure 1f) was immersed in RPMI-1640 chemically defined media and inoculated with green fluorescent protein (GFP) labeled *P. aeruginosa* strain PAO1 (Figure 1g). This bacterial strain was chosen due to its prevalence in biofilms within wound care and both acute ventilator associated lung infections and chronic lung infections in cystic fibrosis.[18–20] The attachment and accumulation of bacteria on each polymer spot was quantified by a fluorescence scanner after 72 h of incubation (Figure 1h). This experiment was repeated twice, with three replicate samples per assay, resulting in two biological replicates and a total of six replicates per sample.

#### 2.1.1 Surface Chemical Analysis

**Figure 2:**
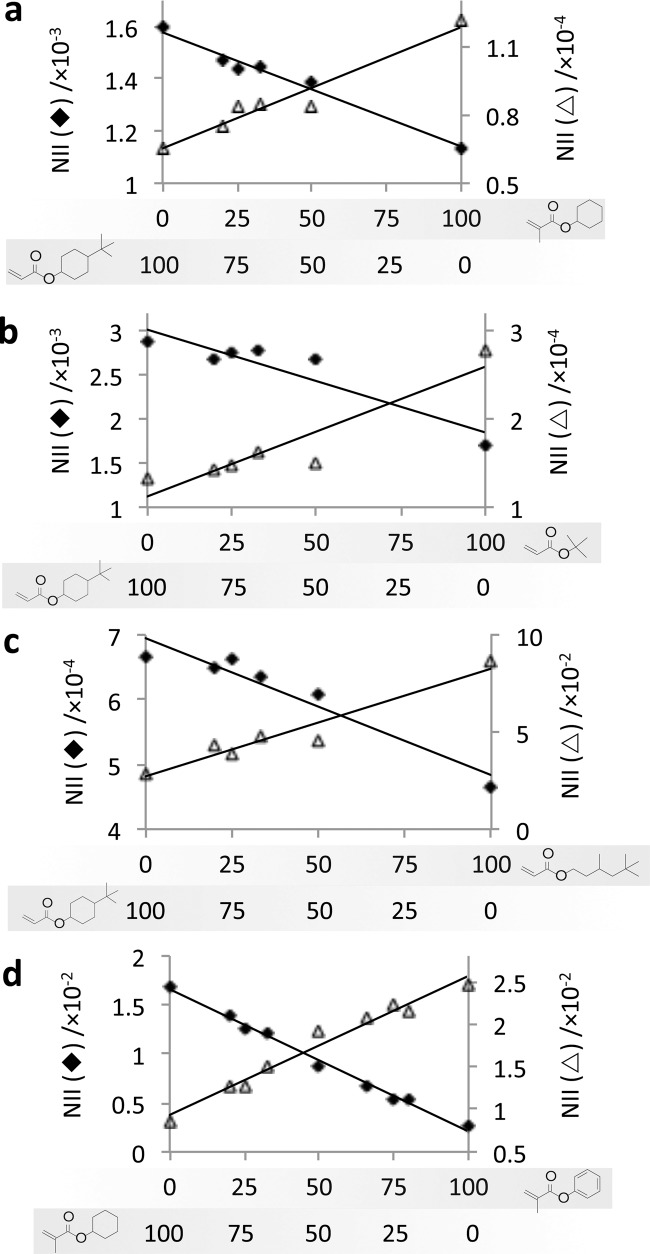
Normalized ion intensity (NII) for representative SIMS ions for copolymers of *t-*BCHA with a) CHMA, b) *t*-BA, and c) TMHA and d) for copolymers of CHMA and BMA. The content (%) of each mono­mer is indicated below each graph. The ions for each graph are a) C_4_H_4_^+^ (♦) indicative of *t-*BCHA (*R*^2^ = 0.97) and C_14_H_28_O_4_^+^ (△) indicative of CHMA (*R*^2^ = 0.95), b) C_4_H_6_^+^ (♦) indicative of *t-*BCHA (*R*^2^ = 0.86) and C_13_H_16_O_4_^+^ (△) indicative of *t-*BA (*R*^2^ = 0.87), c) C_4_H_2_^+^ (♦) indicative of *t-*BCHA (*R*^2^ = 0.93) and C_3_H_7_^+^ (△) indicative of TMHA (*R*^2^ = 0.93), d) C_6_H_11_^+^ (♦) indicative of BMA (*R*^2^ = 0.99) and C_6_H_5_^+^ (△) indicative of CHMA (*R*^2^ = 0.97).

The surface chemistry of each material was assessed across the copolymer sets using ToF-SIMS.[21] Considering the similarities within the pendant groups of the monomers used, SIMS was an ideal technique to detect subtle differences in the chemistry of the materials. The ToF-SIMS spectra contain secondary ions, which are strongly related to the monomers in this study (1Figure a–e). Linear relationships with the intensity of the characteristic ions against commoner composition were observed, as shown in **Figure** **2**. Ions originating from the *tert-*butyl group of *t-*BCHA such as C_4_H_4_^+^ and C_4_H_6_^+^ were found to be representative of *t-*BCHA (Figure 1a), even when mixed with *t-*BA or THMA that also contain a *tert-*butyl group. This suggests that a cyclohexyl linker group between the *tert-*butyl group and the ester group produces a higher yield of C_4_H_4_^+^ and C_4_H_6_^+^ ions than having no linker group (*t-*BA) or an iso-pentyl linker group (TMHA).

The secondary ions most suitable for tracking CHMA and *t*-BA were dimers (Figure 1b,c). CHMA and *t*-BA are structurally very similar to *t-*BCHA with either the *tert-*butyl group or the fragment with cyclohexyl group removed. It is therefore unsurprising that ion fragments produced from CHMA and *t*-BA are also produced from *t-*BCHA such that these ions could not discriminate well between the two monomers. The dimer peaks were able to track CHMA and *t-*BA across the entire series including their respective homopolymers, suggesting that the peaks originate from a pair of CHMA and *t*-BA and not from a dimer that includes *t-*BCHA. The ions most representative of CHMA was C_14_H_28_O_4_^+^ (Figure 1b), while the ion most representative of *t-*BA was C_13_H_16_O_4_^+^ (Figure 1c). The ion C_3_H_7_^+^ was most representative for TMHA. It is likely that this ion originates from methyl side group, which is the most functionally distinct aspect of this monomer compared to *t*-BCHA. For co-polymers of CHMA and CMA, ToF-SIMS was able to discern between the benzyl group, with representative ions such as C_6_H_2_^+^ and C_6_H_5_^+^ (Figure 1e), and the cyclohexyl group, with ions such as C_6_H_11_^+^ and C_6_H_9_^+^ (Figure 1b).

For the copolymers of *t-*BCHA with CHMA or TMHA, the ion intensity trends varied linearly with the bulk concentration (Figure 2a,c, respectively, *R*^2^ > 0.93). For the copolymer of *t-*BCHA and *t-*BA, the intensity of ion C_4_H_6_^+^ characteristic for *t-*BCHA decreased by 17% from 2.9 × 10^−3^ to 2.7 × 10^−3^ as the concentration of *t-*BA was increased from 0% to 50%. A complementary decrease was observed for the ion C_13_H_16_O_4_^+^ characteristic for *t-*BA (Figure 2b). The nonlinear relationship between the change in bulk concentration of the monomer and the intensity of characteristic ion for the monomers may suggest that *t-*BCHA preferentially surface segregates compared with *t-*BA.[22] Alternatively, this may be caused by the increased volatility of *t-*BA due to its lower molecular weight that will alter the monomer composition between printing and photo-curing (typically < 30 s) due to evaporation. This will be more significant for lower bulk concentrations of *t-*BA. For the copoly­mers of CHMA and BMA, the surface concentration of each mono­mer varied closely with changes in the bulk composition (*R*^2^ > 0.97) (Figure 2c). It is unlikely that the changes in the intensity of the representative ions across the copolymer series can be wholly explained by matrix effects[23] as the chemical matrix across a polymer series is similar and the ion intensity trends were observed for multiple representative ions.

#### 2.1.2 Comparison of Surface Chemistry and Bacterial Attachment

**Figure 3:**
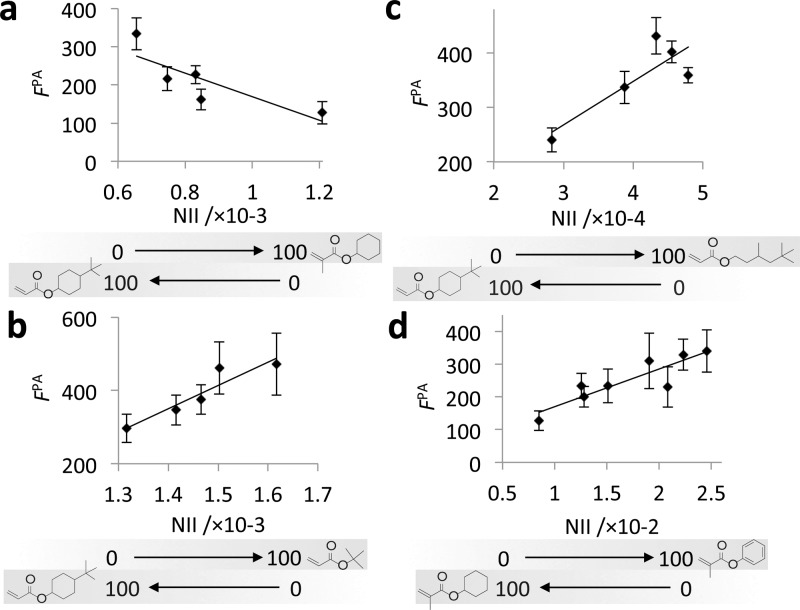
The fluorescence from GFP-labeled *P. aeruginosa* (*F*^PA^) measured on varied copolymer compositions of *t-*BCHA with a comonomer a) CHMA (*R*^2^ = 0.68), b) *t-*BA (*R*^2^ = 0.88), c) TMHA (*R*^2^ = 0.71), and d) for the copolymer of CHMA and BMA (*R*^2^ = 0.76). The surface composition of the polymers is represented by the normalized ion intensity (NII) for ions specific to comonomers, a) C_14_H_28_O_4_^+^, b) C_13_H_17_O_4_^+^, c) C_3_H_7_^+^, and d) C_6_H_5_^+^. Error bars equal ±1 standard deviation unit, *n* = 6.

To assess the relationship between the surface chemistry of the materials and bacterial attachment, bacterial fluorescence was plotted against the chemistry of the copolymer series as represented by the ToF-SIMS normalized ion intensity (NII) in **Figure** **3**. For all copolymer pairs, a near linear trend was observed between *F*_PA_ and the intensity of representative ions (*R*^2^ > 0.68). Inclusion of either of *t-*BA or TMHA without a cyclic hydrocarbon group with the resistant *t-*BCHA resulted in an increase in bacterial attachment (Figure 3b,c). However, the inclusion of CHMA that has a cyclic hydrocarbon group without the *tert-*butyl group with *t-*BCHA resulted in a further decrease in bacterial attachment (Figure 3a). Thus, the monomers that contained a *tert-*butyl group but did not have a cyclic group reduced resistance to bacteria, suggesting the *tert-*butyl group plays little role in achieving resistance to bacterial attachment. In contrast, CHMA that had only a cyclic pendant group and no *tert-*butyl group improved resistance to bacterial attachment, suggesting the cyclohexyl group plays a key role in repelling bacteria. The addition of BMA to CHMA resulted in a linear (*R*^2^ = 0.76) decrease in fluoresence due to bacterial attachment (Figure 3d). This result suggests the inclusion of the aromatic group promotes bacterial attachment relative to the cyclohexyl group. Following from these observations, we were able to rank the monomers in order of their ability to prevent bacterial attachment: CHMA>*t*-BCHA and BMA>*t­*-BA and TMHA.

The materials were also characterized by water contact angle (WCA),[24] as this parameter is often invoked to explain bacterial behavior towards materials.[25,26] Across all copolymer series, there was no variance in WCA, whereupon all materials had a WCA of approximately 90^o^. Inclusion of TMHA resulted in a slight decrease in WCA to 82^o^. The similar WCA of these materials means that differences in their biological performance cannot be explained purely by the surface energy as estimated from the WCA of the materials, although there could be changes in the dispersive surface energy that this method does not probe.

### 2.2 Correlation of Bacterial Attachment with Molecular Descriptors

Generally, we observed in the set of 24 copolymers from 5 monomers that polyacrylates with more cyclic pendant groups exhibited better resistance to bacterial attachment. Cyclic structures are more rigid than linear structures, thus, this result may suggest that increasing molecular rigidity improves resistance to bacterial attachment and that this parameter may generally be applicable for predicting the bacterial response to a material in silico. As a measure of flexibility, the number of rotatable bonds (*n*RotB) for each monomer pendant group was determined, and for copolymers the *n*RotB was determined from the ratio of the two monomer components as predicted from ToF-SIMS. The *n*RotB did not correlate with the fluorescence due to *P. aeruginosa* attachment (*R*^2^ = 0.37). Additionally to molecular flexibility, previous modeling has demonstrated that molecular hydrophobicity plays a key role in bacterial attachment and that, more specifically, the *c*log *P* can be used to predict bacterial attachment[8] and thus was calculated for each monomer.[27] The relative copolymer values were determined based upon the ratio of the monomer components as predicted from ToF-SIMS. Bacterial attachment also did not correlate with *c*log *P* (*R*^2^ = 0.04),[28] such that for the materials used in this study, the interaction with bacteria cannot be explained by assessing molecular polarity or flexibility individually. To investigate the combined effect of molecular polarity and flexibility on bacterial attachment, a composite parameter α was calculated from Equation (1), whereby the coefficient of *n*RotB was optimized to produce the maximum *R*^2^ value between α and fluorescence due to *P. aeruginosa* attachment. The 24 materials formed from the five monomers can be considered as the training set for the determination of α.

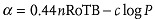
1

**Figure 4:**
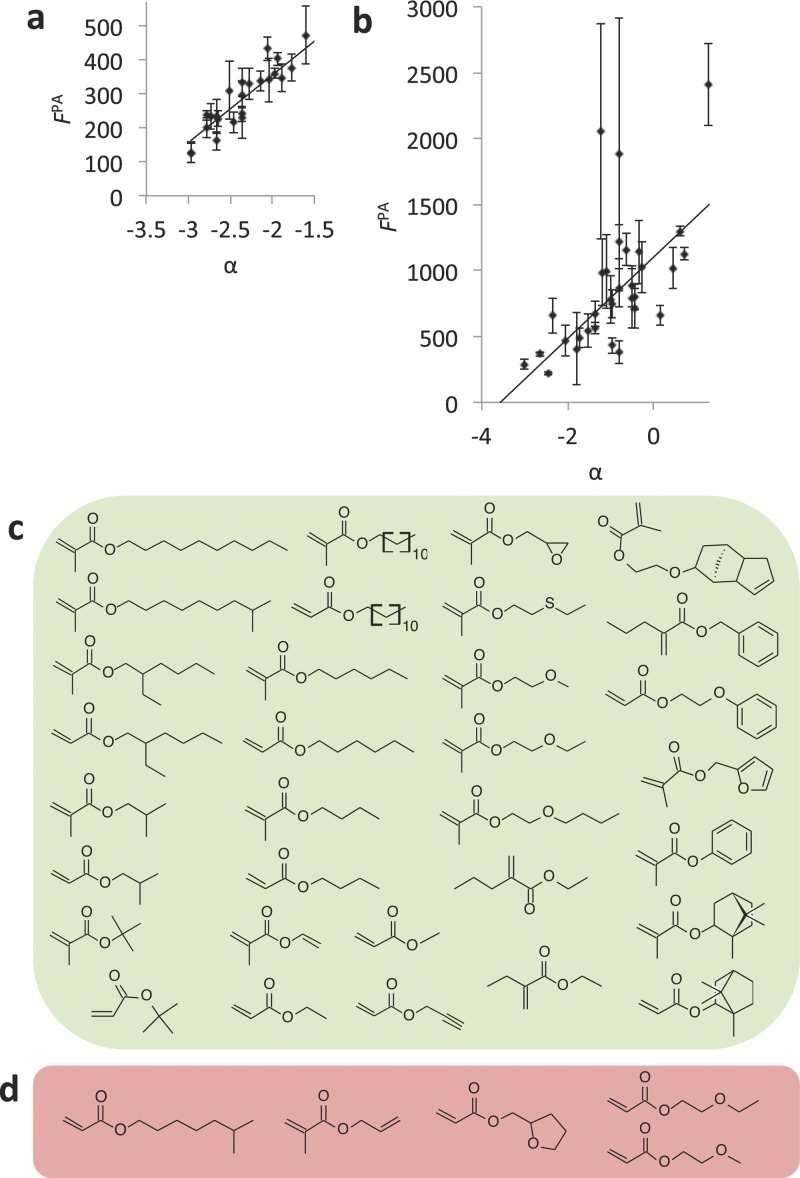
a) Correlation between bacterial attachment with molecular flexibility and polarity. The fluorescence due to the attachment of *P. aeruginosa* (*F*^PA^) is plotted against the composite parameter α (*R*^2^ = 0.83) for each polymer used in this study. Error bars equal ±1 standard deviation unit, *n* = 6. b) Correlation between α and *F*^PA^ on 37 homopolymers (*R*^2^ = 0.67). Two samples with *F*^PA^ above 3000 that were unsuccessfully correlated with α are not shown to enable better visualization of those samples where a correlation was observed. The coordinates of these two samples are (1.2, 7700) and (0.1, 14600). Error bars equal ±1 standard deviation unit, *n* = 3. c,d) Chemical structures of the monomers of polymers whereby c) the bacterial attachment was successfully correlated with α, and d) higher bacterial attachment was measured than what was expected from assessing α.

The parameter *α* correlated with bacterial attachment with *R*^2^ = 0.83 (**Figure** **4**a). This result suggests that low bacterial attachment is observed on materials that are both nonpolar and are molecularly rigid. A previous partial least square (PLS) model correlated the attachment of *P. aeruginosa* to ToF-SIMS ions with *R*^2^ = 0.68,[7] while a nonlinear model correlated the same bacterial data with molecular descriptors with *R*^2^ = 0.87.[8] The training set represents a much smaller chemical diversity than the materials used in these two other models; nevertheless, the present model achieved an excellent correlation between bacterial attachment and a material property compared with previous models.

To test the robustness of the model, it was applied to a dataset of bacterial attachment previously acquired on a library of all commercially available (meth)acrylate monomers polymerized as a polymer microarray.[6] Initially, monomers including fluorocarbon, hydroxyl, amine, or multiple glycol moieties and di- or tri-acrylates were unsuccessfully modeled. Considering the chemical space represented in the training set, it is unlikely that the model will be predictive for materials that do not have hydrocarbon pendant groups. Thus, these materials were excluded from further analysis and α was calculated for all the (meth)acrylate materials containing hydrocarbon pendant groups. The resultant α values were normalized to the bacterial response in the initial training sets for samples represented in both the training and test sets. This was necessary due to the biological variance between different experiments. In total, the model was applied to 37 samples, most of which represented chemistries outside those represented in the training set for the model (Figure 4b). A correlation between *P. aeruginosa* attachment and *α* was observed for 32 samples (*R*^2^ = 0.67). Thus, *α* was predictive for bacterial attachment for materials outside the training set but within the chemical space of poly(meth)acrylates with hydrocarbon pendant groups. For five samples (Figure 4d), the bacterial response was higher than what was expected by assessing *α*. High biological variance associated with two of the measurements likely limits the application of the model to these samples. As the training set for α was based upon material with low bacterial attachment, it is likely that α does not fully capture surface–biological interactions that promote bacterial attachment. It is, therefore, unsurprising that some samples exhibit higher bacterial attachment than is predicted from α. Specifically, the interaction of *P. aeruginosa* with ethylene glycol and tetrahydrofurfuryl moieties is not fully captured by α. Nevertheless, there is a broad range of hydrocarbon chemistries outside those represented in the training set whereupon α successfully correlated with the experimentally measured *P. aeruginosa* attachment, suggesting that α is predictive of bacterial attachment over a broad chemical space. To our knowledge, this is the most successful extrapolation of a model of bacterial attachment based upon molecular descriptors to a sample set of polymers. In particular, α correlates well with polymers associated with low bacterial attachment and is, therefore, likely an excellent tool for the further development of materials able to resist bacterial attachment.

The reduced bacterial attachment observed on the weakly amphiphillic poly(meth)acrylates has not been limited to a single pendant group nor to a single bacterial strain, including both Gram-negative and Gram-positive species,[6,7] suggesting that the interaction with the polymers' pendant groups would not involve a specific signaling pathway but rather is associated with a bacterial property that is common to multiple strains/species. In the present study having a pendant group with both a nonpolar nature and increased rigidity has been correlated with decreased bacterial attachment. This may suggest an interaction with the bacterial membrane, whereby an increase in the rigidity of the side group may more effectively disrupt or apply strain to the membrane triggering a biochemical signaling response resulting in nonattachment. No evidence for a killing action of these polymers has been observed,[7] so the membrane interaction may result in a cell-signaling mechanism that prevents bacterial attachment.

## 3. Conclusion

Biofilm formation is a key problem for the clinical use of medical devices as we enter the post-antibiotic era. New materials that are able to resist bacterial attachment will greatly enhance the performance of medical devices and reduce mortality and morbidity. In this study, 24 copolymers were prepared to compare linear, cyclic, and aromatic side groups on the attachment of *P. aeruginosa*. A cyclic pendant group achieved a greater reduction in bacterial attachment compared with either an aromatic or linear pendant group. A parameter combining *c*log *P* and rRotB together produced a strong correlation with bacterial attachment (no correlation observed individually). This parameter was successful in correlating molecular flexibility and hydrophobicity with bacterial attachment for a broad range of hydrocarbons, beyond those used to train the composite para­meter. This suggests that bacteria are repelled from a surface-exhibiting rigid hydrophobic chemical moieties in the specific case of poly(meth)acrylates with hydrocarbon pendant groups. We note that for these polymers the hydrophobic groups will always be partnered with a hydrophilic ester group, hence, the materials will present rigid amphiphilic moieties. This insight will help inform the further development of polymers resistant to bacterial attachment.

## 4. Experimental Section

*Polymer Microarray Formation*: For dip-coating, epoxy-coated glass slides (Genetix) were dipped into a 4% (w/v) poly(hydroxyethyl methacrylate) (pHEMA) solution in ethanol and withdrawn at a rate of 30 mm s^−1^. Slides were then inverted and then maintained in a near horizontal position for 10 min before being left to dry at ambient conditions for a week in a slide holder. Polymer microarrays were formed as previously described.[10,29] Briefly, microarrays were prepared using a XYZ3200 dispensing workstation (Biodot). Printing conditions were O_2_< 1300 ppm, 25 °C, 30–40% humidity. Slotted metal pins (946MP6B, Arrayit) were used to transfer approximately 2.4 nL of monomer solution onto 10 pHEMA-coated substrates before slides were irradiated with a long wave UV source for 30 s. The monomer solutions were composed of 75% (v/v) monomer in DMF with 1% (w/v) photoinitiator 2,2-dimethoxy-2-phenylacetophenone. Once formed arrays were dried at <50 mTorr for 7 d.

*Water Contact Angle Measurements*: Sessile WCA measurements were taken of each polymer present on the array. Ultrapure (18.2 MΩ resistivity at 25 °C), filtered (0.2 μm) water was injected onto each spot using a Drop Shape Analyzer 100 (Krüss). 1 drop was deposited per spot using a voltage of 60 V and a pulse width of 100 μs, giving an average spot volume of 410 pL. 20× magnification images of the spots evaporating were recorded and the contact angle was determined from the first frame showing the spot upon the surface. *c*log P was calculated using ACD/ChemSketch V14.01 Software (ACD/Labs).

*Time-of-Flight Secondary Ion Mass Spectrometry*: Measurements were conducted using an IONTOF IV ToF-SIMS instrument operated using a 25 kV Bi_3_^+^ primary ion source exhibiting a pulsed target current of ≈1 pA. The primary ion beam was rastered over analysis areas of 100 μm × 100 μm. An ion dose of 2.45 × 10^11^ ions cm^−2^ was applied to each sample area ensuring static conditions were maintained throughout. Both positive and negative secondary ion spectra were collected (mass resolution of >7000), over an acquisition period of 15 scans (the data from which were added together). Owing to the nonconductive nature of the samples, charge compensation, in the form of a low energy (20 eV) electron floodgun, was applied.

*Bacteria and Growth Conditions*: *P. aeruginosa* PAO1 was routinely grown on either LB (Luria-Bertani, Oxoid, UK) agar plates at 37 °C or in broth at 37 °C with 200 rpm shaking. The GFP-expressing plasmid, pGFP[7] was transformed into *P. aeruginosa* PAO1 by electroporation and maintained by adding the appropriate antibiotics to the culture medium. RPMI-1640 chemically defined medium (Sigma, UK) was used in biofilm experiments. Prior to incubation with the bacteria, the microarray slides were washed in distilled H_2_O for 10 min, air-dried, and UV sterilized. Bacteria were grown on polymer slides under similar conditions to those previously described.[30,31] Briefly, UV-sterilized polymer slides were incubated in 15 mL medium inoculated with diluted (OD_600_ = 0.01) GFP-tagged bacteria from overnight cultures grown at 37 °C with 60 rpm shaking for 72 h. As growth medium controls, the slides were also incubated without bacteria. At the desired time points, the slides were removed and washed three times with 15 mL phosphate buffer saline at room temperature for 5 min. After rinsing with distilled H_2_O to remove salts, the slides were air dried. Fluorescent images from the slides incubated in medium only and medium containing bacteria were acquired using a GenePix Autoloader 4200AL Scanner (Molecular Devices, USA) with a 488-nm excitation laser and a blue emission filter (510–560 nm). The total fluorescence intensity from polymer spots was acquired using GenePix Pro 6 software (Molecular Devices, USA). The fluorescence signal (*F*) from each bacterial pathogen was determined by subtracting the fluorescence from the slide incubated in media only from the fluorescence after incubation with bacteria.
